# Netrin-1 plays a role in the effect of moderate exercise on myocardial fibrosis in rats

**DOI:** 10.1371/journal.pone.0199802

**Published:** 2019-02-21

**Authors:** Zhou Daliang, Yu Lifang, Fu Hong, Zhang Lingling, Wei Lin, Li Dapeng, Zhang Tianshu, Li Weimin

**Affiliations:** 1 Department of Cardiology, First Hospital of Harbin City, Harbin, Heilongjiang Province, China; 2 Clinical Pharmacy, First Affiliated Hospital, Heilongjiang University of Chinese Medicine, Harbin, Heilongjiang Province, China; Max Delbruck Centrum fur Molekulare Medizin Berlin Buch, GERMANY

## Abstract

**Introduction:**

This study aimed to investigate the effect of aerobic exercise on the expression of neitrin-1,DCC receptor and myocardial fibrosis in rats with acute myocardial infarction.

**Methods:**

Twenty-four rats were randomly divided into three groups: the sham group (n = 8), the acute myocardial infarction (AMI) model group (n = 8), and the aerobic exercise treatment after acute myocardial infarction group (ET) (n = 8). After 10 weeks, the serum levels of netrin-1, tumor necrosis factor alpha α (TNF-α), and interleukin 6 (IL-6) were measured. The expression of matrix metalloproteinase 2 and 9 (MMP2, 9), and their inhibitor, tissue inhibitor of metalloproteinase 2 (TIMP2), myocardial netrin-1, and the deleted in colorectal cancer (DCC) receptor were evaluated. Histopathological results were also evaluated. The collagen volume fraction of the myocardial tissues was also calculated.

**Results:**

Compared with the sham group, in the AMI and ET groups, left ventricular end diastolic pressure (LVEDP) were increased, while left ventricular systolic pressure (LVSP), and left ventricular pressure maximal rate of rise and fall (± dp/dtmax) were significantly decreased (P<0.05,). Compared with the AMI group, in the ET group, LVSP, and ±dp/dtmax were significantly increased while LVEDP was decreased (P<0.05). Compared with the sham group, the AMI group and ET groups showed increased levels of serum TNF-α, IL-6 and significantly reduced levels of netrin-1. Levels of TNF-α and IL-6 were significantly reduced in the ET group compared with the AMI group, whereas the level of netrin-1 was increased. The expression of myocardial MMP2 and MMP9 was significantly increased in the AMI group compared with the sham group, whereas that of myocardial netrin-1, TIMP2 and the DCC receptor, was significantly reduced. Compared with the AMI group, the ET group showed reduced expression of myocardial MMP2 and MMP9 proteins, whereas expression of myocardial netrin-1, TIMP2 and the DCC receptor, was significantly increased. The collagen volume fraction of the myocardial tissues was significantly increased in the AMI group and the ET group compared with the sham group, with a greater increase in the AMI group.

**Conclusions:**

Aerobic exercise increased levels of serum netrin-1, myocardial netrin-1, and the DCC receptor and reduced the expression of myocardial MMP2 and MMP9 proteins, to improve the degree of fibrosis following myocardial infarction in rats.

## Introduction

Despite modern medical advances, acute myocardial infarction is still associated with high morbidity and mortality. In the weeks following myocardial infarction, the necrotic myocardium is gradually replaced with scar or fibrous tissue, during the healing process. Scarring helps to maintain the structure of the heart, and prevents myocardial rupture [1]. However, fibrous tissue can also cause the loss of myocardial systolic function, and excessive myocardial fibrosis can induce changes in the shape and function of the heart, a phenomenon referred to as ventricular remodeling. These changes can eventually lead to the development of severe heart failure that can accelerate the death of patients [2–4]. Proper control of the degree of myocardial fibrosis is very important to prevent fibrosis of the non-infarcted zone.

The role of netrin-1 in cardiovascular disease and the stages of acute inflammation is an emerging area of research. Some studies have shown that an appropriate increase in the concentration of netrin-1 can alleviate myocardial ischemia-reperfusion injury, and reduce atherosclerosis [5,6].

Some studies have shown [6,7] that netrin-1 can activate downstream p44/42 mitogen-activated protein kinases and endothelial nitric oxide synthase (eNOS), and induce NO production that protects against infarction-induced ischemic injuries, and reduce the infarct area, by binding to the deleted in colorectal cancer (DCC) receptor. When the DCC gene is knocked out, this effect is weakened.

Aerobic exercise has shown positive results in improving the poor prognosis of patients with myocardial infarction. Previous studies have shown that aerobic exercise can improve heart function in patients with myocardial infarction, reduce the area of myocardial necrosis, and reduce the degree of ventricular remodeling with low risks [3]. However, many of the associated mechanisms remain unclear.

Liu and others [8] have reported that aerobic exercise could increase the expression of netrin-1 to relieve cerebral reperfusion injury in rats. However, whether aerobic exercise has similar effects in myocardial cells has not yet been reported.

Therefore, the present study was undertaken to investigate the changes in netrin- 1 expression after myocardial fibrosis, and evaluate the effect of aerobic exercise on netrin-1 after myocardial infarction.

## Materials and methods

### Animals and groups

Animal experiments were approved by the animal committee of Harbin medical university(SCXK HEI 201605). Healthy adult male Sprague–Dawley rats, weighing 180–220 g were supplied by the Animal Experiment Center of Harbin Medical University. The rats were randomly divided into the acute myocardial infarction (AMI) model group (n = 8) and the aerobic exercise treatment after acute myocardial infarction group (ET) (n = 8), following the production of an anterior wall myocardial infarction (described in the following section). Another eight rats were allocated to a sham group. All animals were allowed to adapt to the environment in the laboratory over a period of 1 week. All animals were immediately euthanized by intraperitoneal injection of 200 mg/kg sodium pentobarbital after 10weeks.

### Myocardial infarction procedure

Rats in the AMI and ET groups were subjected to permanent ligation of the left anterior descending coronary artery (LAD), using a 6–0 suture to induce myocardial infarction [9]. Under deep anesthesia [ketamine (10 mg/kg body weight)], the thorax was incised at the third intercostal space, and the LAD was ligated 2 mm below the left atrium. Occlusion of the LAD was confirmed microscopically through discoloration of the ischemic area below the ligature. The intercostal space, muscle layers, and skin were closed using continuous sutures with 6–0 silk. Sham animals underwent a similar procedure without occlusion of the LAD.

### Exercise training protocol

At 1 week after myocardial infarction, rats of the ET group began to adapt to training on a rat treadmill. The speed was set at 10–12 m/min, and rats were initially placed on the treadmill for 10 min every day, for 3 days. After this period of adaptation, the speed was increased to 16 m/min (60% exhaustion exercise), at a grade of 0°, for 40 min every day, 5 days a week, over 10 weeks [10].

#### Exhaustion exercise

According to the methods from literatures[11], a little change is made.At 2 days after training, the load and movement of the rats were increased. The following protocol was applied: running at approximately 5 m/min, 10 min after warming up, followed by a starting load at 10 m/min, with a gradual increase over 3 min, followed by 5 m/min, until exhaustion. The following criteria were used to determine whether the animal was exhausted: inability of the animal to keep up with a predetermined speed; hip pressure of the rat against the back wall of the cage while on the treadmill; dragging of the hind legs for 30. Any stimulation of the rats to drive them forward invalidated the determination of exhaustion.

### Left ventricle function assessment

After 10 weeks, the rats were anesthetized using an intraperitoneal injection of chloral hydrate (300 mg/kg), The rats underwent left ventricular intubation through the right common carotid artery, Connect the pressure sensor (ap-621g carrier amplifier) and the rm-6000 8-channel physiological recorder, that records left ventricular systolic pressure (LVSP), left ventricular end-diastolic pressure (LVEDP), and left ventricular pressure maximal rate of rise and fall (±dp/dtmax).

### Determination of Serum of IL-6, netrin-1, and TNF-α

Serum interleukin 6 (IL-6), netrin-1, and tumor necrosis factor alpha α (TNF-α) were quantified using a commercially available enzyme-linked immunosorbent assay (Cusabio Biotech) following the manufacturer’s protocol. Blood was collected via the abdominal aorta, and samples were centrifuged for 10 min at 10,000 *g* and 4°C, and stored at -80°C until further analysis.

### Western blot analysis

After 10 weeks, myocardial tissue was harvested, including necrotic myocardial tissue and healthy myocardial tissue from 5 mm outside the area of necrotic myocardium. The expression of myocardial matrix metalloproteinases 2 and 9 (MMP2, 9) proteins, tissue inhibitor of metalloproteinases 2 (TIMP2), netrin-1 and DCC receptor was measured as follows. Proteins were extracted from all experimental samples and separated through electrophoresis on 8%, 10%, or 12% polyacrylamide gel containing sodium-dodecyl sulfate gel, and then transferred onto a polyvinylidene difluoride membrane. The membranes were incubated for 1 h at room temperature, in 5% nonfat dry milk in Tris-buffered saline solution with Tween 20 (TBST), to block any non-specific binding sites. Membranes were then incubated overnight at 4°C in primary antibodies diluted in a 5% bovine serum albumin solution in TBST. The antibodies used for western blot analysis were specific for the following proteins: MMP2(Abcam,1;1000), MMP9(Abcam,1:1000),TIMP2(Abcam,1:500),netrin-1(Abcam,1:500),DCCreceptor(Biorbyt,1:100),and GAPDH (1:2000). Membranes were subsequently incubated with secondary antibodies for 1 h at room temperature and developed using an enhanced chemiluminescent detection system. The films were scanned and analyzed using Bio-Rad Image Lab software. All experiments were repeated more than three times.

### Histopathology

After fixation, dehydration, and embedding in paraffin, hematoxylin and eosin (H&E) and Masson staining was performed on the myocardial tissue. Masson staining renders myocardial cells red, and collagen blue. Motic Med 6.0 software was used for image analysis and measurement of the myocardial collagen volume fraction (CVF). The CVF = myocardial collagen area/test vision area, and values of this parameter were randomly selected from each of six tissue sections. The average of 10 visual measurements from each randomly selected tissue section was recorded.

### Statistical analysis

Results were expressed as the mean ± standard error. Data were submitted to *t*-test (two groups) or one-way ANOVA (three groups).Statistical analyses were performed using SPSS 11.0 software. P<0.05 was considered a statistically significant difference.

## Results

### Left ventricle function comparison

**Fig 1:**
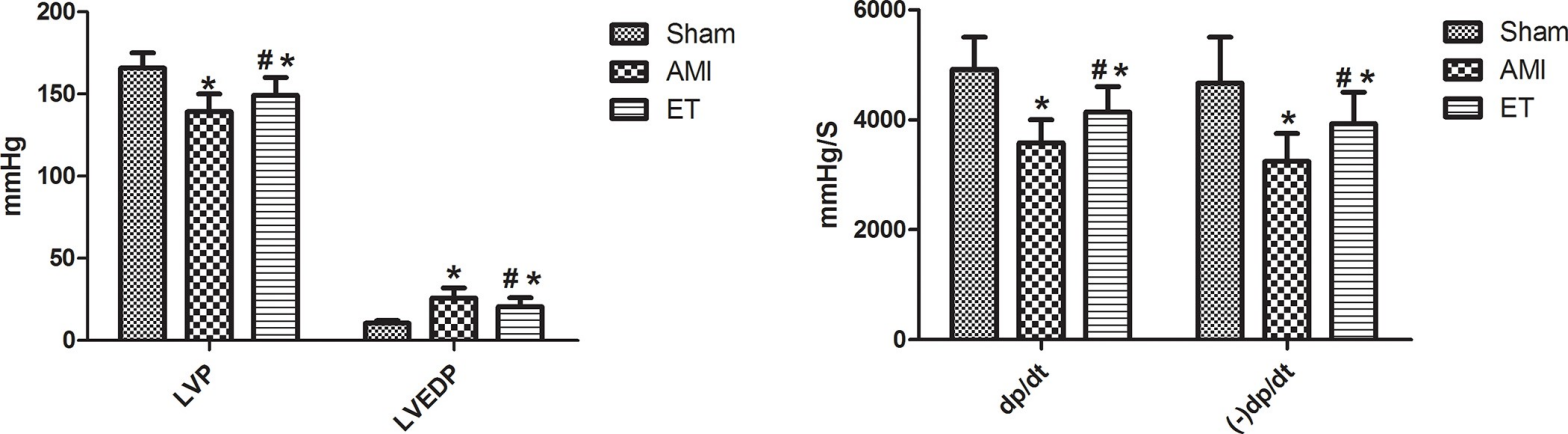
Left ventricle function assessment. *P<0.05 versus sham; #P<0.05 versus the AMI group;LVEDP,left ventricular end diastolic pressure;LVSP,left ventricular systolic pressure;± dp/dtmax,left ventricular pressure maximal rate of rise and fall;Sham,the sham group (n = 6);AMI,the acute myocardial infarction model group (n = 6);ET,the aerobic exercise treatment after acute myocardial infarction group (n = 6).

Compared with the sham operation group, LVEDP was significantly increased in the AMI group and the ET group, while LVSP, +dp/dtmax and -dp/dtmax were significantly decreased (P<0.05). Compared with the AMI group, +dp/dtmax and -dp/dtmax in the ET group were significantly increased and LVEDP was significantly decreased, (P<0.05, Fig 1).

### Effect of aerobic exercise on myocardial fibrosis after myocardial infarction

**Fig 2:**
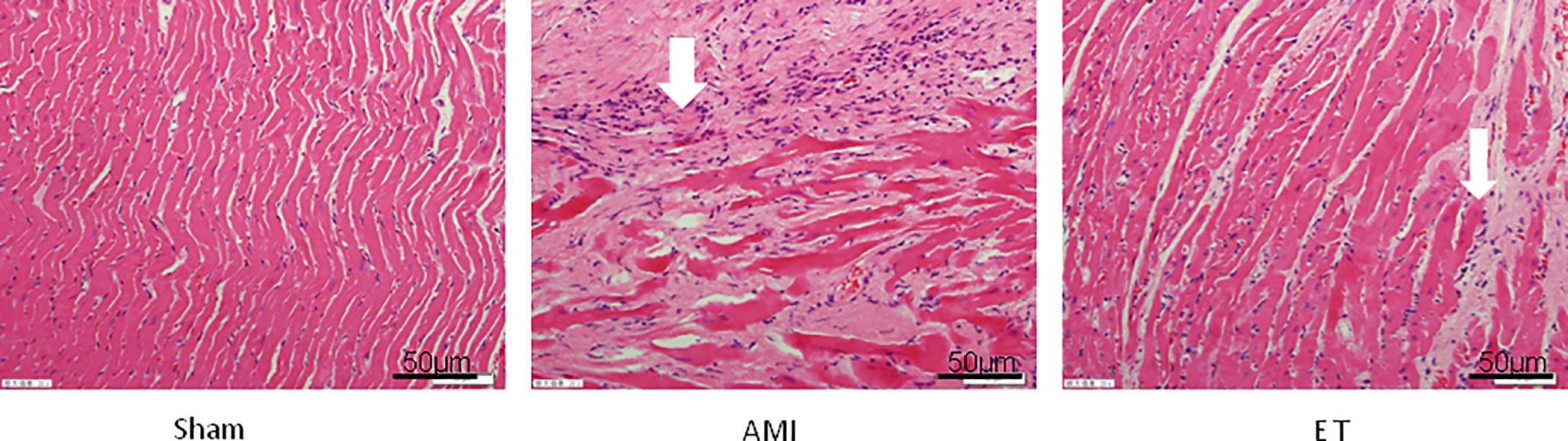
H&E staining (magnification, ×200) of tissues demonstrated that the myocardial cells in the sham group showed good continuity, regular arrangements, Meanwhile, the myocardial cells of AMI,ET rats demonstrated significant hyperplasia and hypertrophy, disordered arrangement, sparseness, swollen cytoplasm, and increased myocardial cell gap.Bar indicates 50 μm.

**Fig 3:**
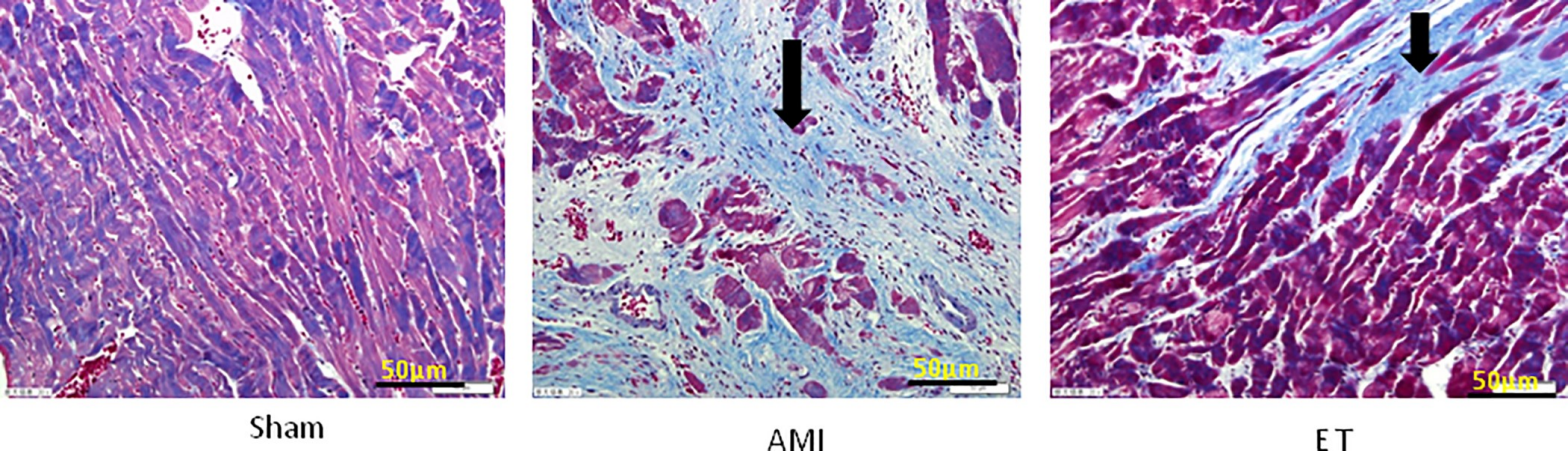
Masson staining was performed on the myocardial tissue(magnification,×200),different types of fibrosis. Rats cardiac tissue was stained with Masson’s trichrome stain to visualize cardiomyocytes in red and fibrotic fibers in blue. Bar indicates 50 μm.

**Fig 4:**
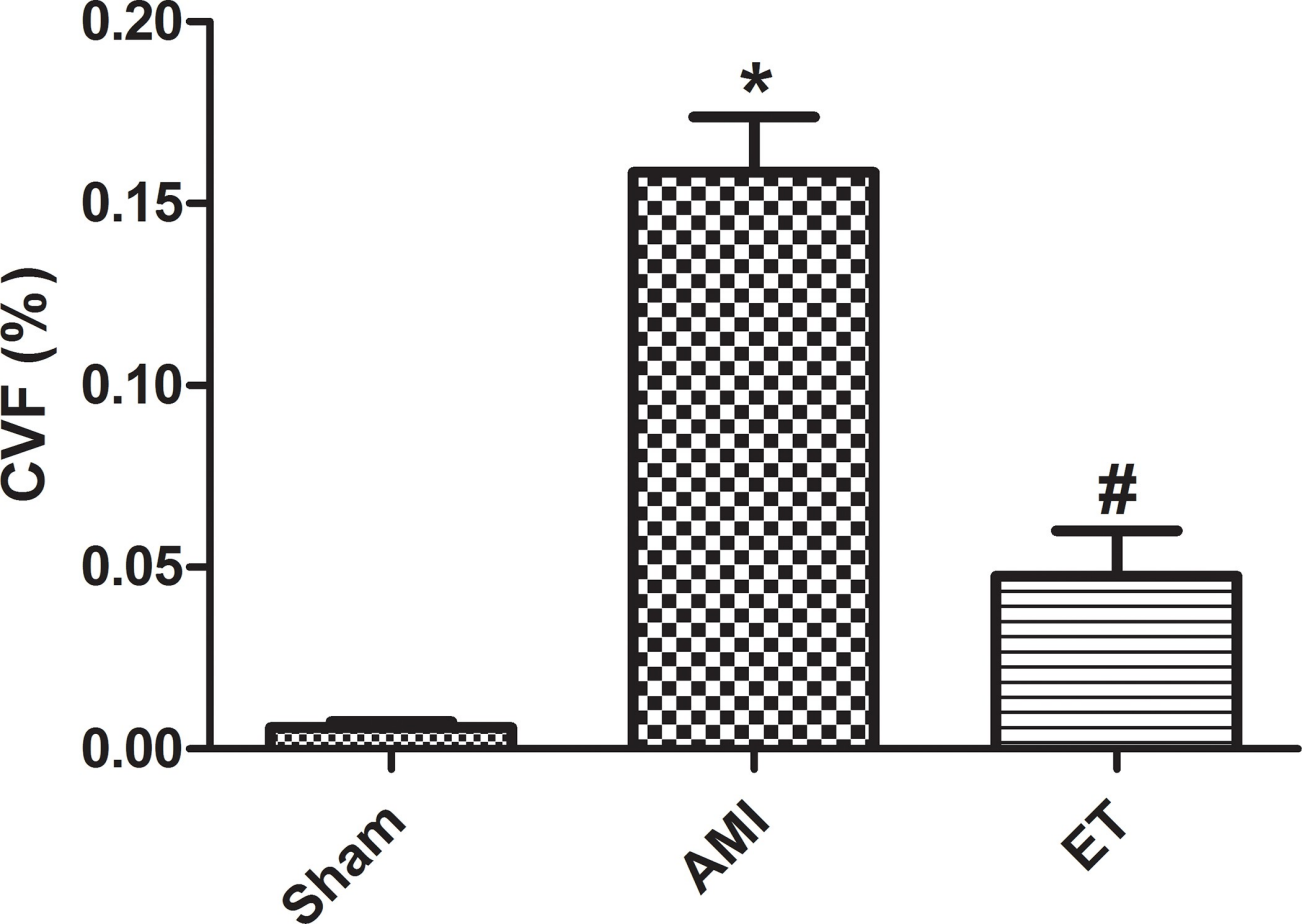
Motic Med software was used for image analysis and measurement of the myocardial collagen volume fraction. *P<0.05 versus sham;#P<0.05 versus the AMI group;;Sham,the sham group (n = 6);AMI,the acute myocardial infarction model group (n = 6);ET,the aerobic exercise treatment after acute myocardial infarction group (n = 6).CVF = myocardial collagen area/test vision area.

With the exception of the sham group, the H&E stained tissues of each group of rats showed varying degrees of inflammatory cell infiltration and cell necrosis, with the most considerable infarction noted in the AMI model group (white arrow, **Fig 2**). Masson staining revealed a large number of blue collagen fibers in the AMI group and significantly less in the ET group (black arrow, **Fig 3**). Compared with the sham group, the AMI and ET groups showed a significantly higher CVF (P<0.05). However, the AMI group showed a more significant CVF increase than that of the ET group (P<0.05, **Fig 4**).

### Effect of aerobic exercise on IL-6, netrin-1, and TNF-α

Compared with the sham group, the AMI and ET groups showed significantly higher serum levels of IL-6, netrin-1, and TNF-α (P<0.05). Compared with the AMI group, the ET group showed significantly lower levels of IL-6, netrin-1, and TNF-α (P<0.05, **Table 1**).

**Table 1:** Changes in the serum of TNF-α,IL-6,Netrin-1 among groups.

Group	n	TNF-α (pg/mL)	IL-6 (pg/mL)	Netrin-1 (pg/mL)
Sham	8	18.17 ± 1.12	100.48 ± 6.65	89.51 ± 6.98
AMI	8	25.89 ± 4.20^a^	161.81 ± 14.29^a^	73.96 ± 12.67^a^
ET	8	22.16 ± 0.79^a^^b^	123.04 ± 28.65^a^^b^	104.78 ± 31.13^a^^b^

AMI, ET vs. Sham

^a^P < 0.05; Sham, ET vs. AMI

^b^P < 0.05; AMI, acute myocardial infarction; ET

aerobic exercise treatment;TNF-α,tumor necrosis factor alpha α; IL-6,interleukin 6.

### Effect of aerobic exercise on protein expression

**Fig 5:**
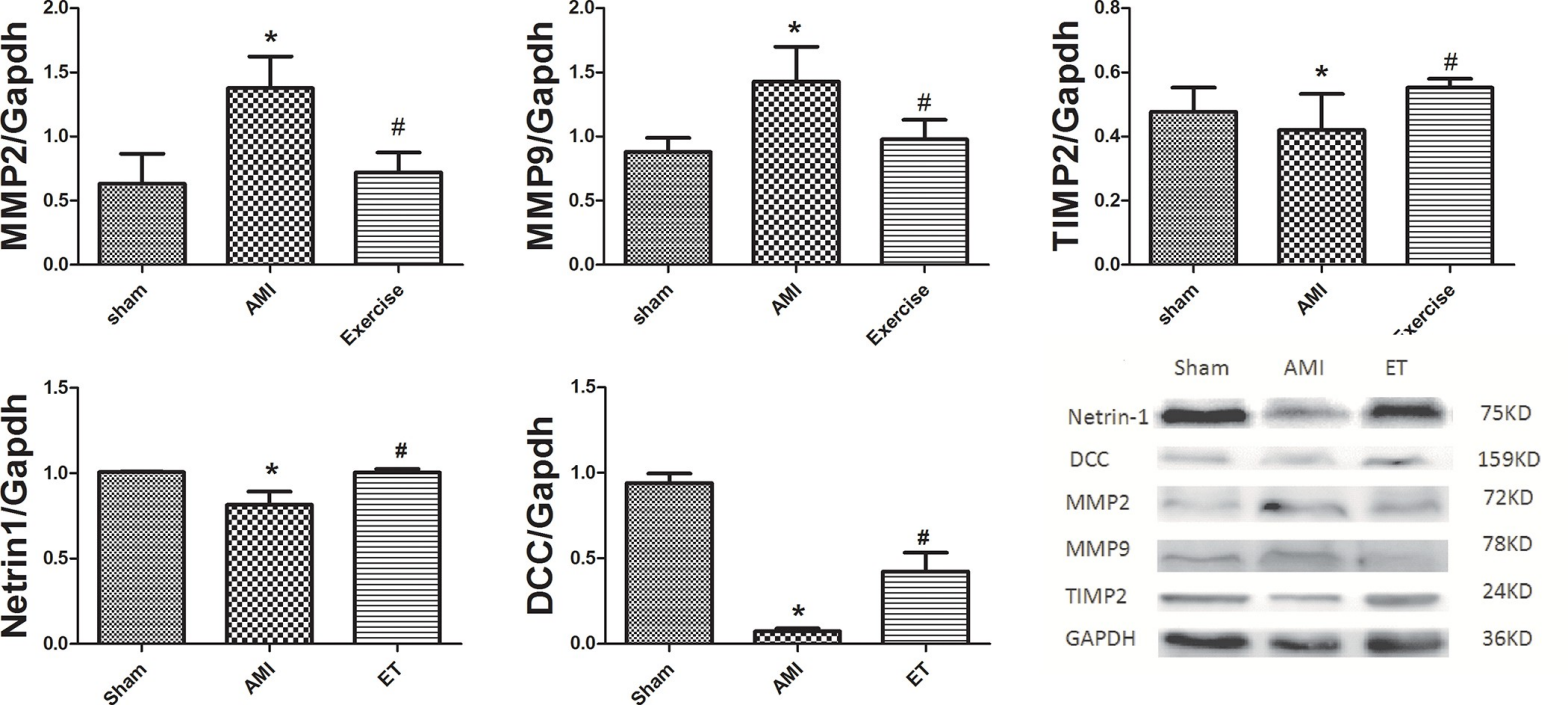
Effect of aerobic exercise on protein expression. *P<0.05 versus sham;#P<0.05 versus the AMI group;Sham,the sham group(n = 3);AMI,the acute myocardial infarction model group(n = 3);ET,the aerobic exercise treatment after acute myocardial infarction group(n = 3);MMP2, 9,myocardial matrix metalloproteinases 2 and 9proteins; TIMP2,tissue inhibitor of metalloproteinases 2;DCC,the deleted in colorectal cancer.

Compared with the sham group, the AMI group and ET group showed significantly higher expression of myocardial MMP2 and MMP9, and reduced expression of myocardial netrin-1, DCC and TIMP2. Compared with the AMI group, the ET group exhibited significantly reduced expression of MMP2 and MMP9 (P<0.05), and significantly increased expression of netrin-1, DCC and TIMP2 (P<0.05, **Fig 5**).

## Discussion

Myocardial fibrosis is the excessive deposition of extracellular matrix (ECM), such as collagens and fibronectin, The aim is repair of damaged tissues,but an excessive deposition of ECM proteins, especially collagens, leading to a pathological remodeling with increased myocardial stiffness, heart failure[12].

Exercise training which is a safe therapeutic tool for the treatment of AMI promotes beneficial effects, such as decreased AMI-induced cardiomyocyte apoptosis, decreased risk of heart failure, and improved cardiac function,but the underlying molecular mechanism is still unclear[13].

The current study found that compared with the sham group, in the AMI and ET groups, LVEDP was increased, while LVSP and ±dp/dtmax were significantly decreased. This indicated that there were different degrees of damage to cardiac function in the AMI group and the ET group, but the damage was relatively minor in the ET group In addition, the H&E-stained tissue samples showed varying degrees of inflammatory cell infiltration and cell necrosis in the AMI and ET groups, but the most considerable infarction-related necrosis and inflammation were observed in the AMI group. Masson staining revealed many blue collagen fibers in samples from the AMI and ET groups, but there were less in the ET group. Therefore, this study successfully established a model of myocardial fibrosis following myocardial infarction in rats and found that aerobic exercise can reduce the degree of myocardial fibrosis and improve cardiac function.

### Effect of aerobic exercise on serum netrin-1, myocardial netrin-1 protein and DCC receptors after myocardial infarction

Netrins are a group of highly conserved secretory proteins that guide cell migration and direct axons. Netrin-1 is part of the Netrin family, is similar to laminin in structure and is believed to be derived from the laminin Y chain. Netrin-1 derives its activity by binding to DCC and neogenin receptors, and mediates repulsion via the uncoordinated-5 (UNC5) receptors [14, 15].

netrin-1 has received a lot of attention in the cardiovascular field in recent years. Some studies have shown that an appropriate increase in the concentration of netrin-1 can alleviate myocardial ischemia-reperfusion injury and reduce atherosclerosis [5, 6]. It has also been shown that the DCC ERK1 netrin-1/2/eNOS feed-forward mechanism stimulates aortic endothelial cells to produce NO to protect myocardial cells [16]. This is a key mechanism, through which regulation of eNOS results in NO production and myocardial cell protection in acute myocardial ischemia, chronic congestive heart failure, and ventricular fibrosis [5]. It has also been reported that netrin-1 can regulate morphological changes in endothelial cells and vascular smooth muscle cells.[17].

In the current study, we found that after 10 weeks the AMI group showed reduced levels of serum netrin-1 and reduced expression of netrin-1 protein and DCC receptors in myocardial tissues, whereas the ET group showed significantly increased expression of myocardial netrin-1 protein, DCC receptors and increased levels of serum netrin-1. Therefore, we hypothesized that serum and myocardial netrin-1 expression were decreased after myocardial infarction. However, more importantly, aerobic exercise is likely to improve the degree of myocardial fibrosis after myocardial infarction by increasing the expression of serum and myocardial netrin-1, and this protective mechanism may involve binding of the DCC receptor with netrin-1.

### The effect of aerobic exercise on IL-6, TNF-α, MMP2, MMP9, and TIMP after myocardial infarction

Myocardial fibrosis after myocardial infarction is an important healing process. Myocardial infarction affecting a relatively large area following the necrosis of myocardial cells activates inflammatory cells. The role of inflammatory cell necrosis is to remove cell debris from the myocardium and facilitate the reorganization of the extracellular matrix. However, the levels of many inflammatory cytokines involved in this process, such as IL-6 and TNF-α, become increased, thereby promoting the activation of inflammatory cells [2]. Moreover, excessive activation of inflammatory factors disrupts the stability of the extracellular matrix environment, and further promotes the excessive activation of MMPs, thereby accelerating the process of fibrosis after myocardial infarction, and subsequent deterioration of cardiac function [18, 19].

Considerable collagen deposition occurs following AMI, because of myocardial cell reconstruction and reconstruction of the extracellular matrix in the infarcted area. Significant hyperplasia of collagen positive cells serves to prevent myocardial rupture, and is a natural process of self-protection. However, this process can also increase myocardial stiffness in the infarcted area and reduce its compliance, thereby causing cardiac diastolic dysfunction or failure [1, 20]. MMPs are secreted proteins that are produced mainly by neutrophils, macrophages, smooth muscle cells, and endothelial cells. MMPs play a role both in matrix degradation and in the regulation of collagen synthesis. The inhibitor of MMPs, TIMPs, regulates inhibition under physiological conditions, to maintain a dynamic balance between the actions of MMPs and TIMPs. This effectively coordinates the degradation and reconstruction of the extracellular matrix, and maintains the integrity of its organizational structure and stability of the internal environment [21]. Among the MMP family, MMP2 and MMP9 are two important members associated with the modulation of myocardial fibers, and their activity often causes both increased levels of collagen and the aggravation of fibrosis; however, a decline in their activity often leads to reduced fibrosis [22].

In the current study, we found that 10 weeks after myocardial infarction in rats, levels of serum IL-6 and TNF-α were significantly increased. Different levels of collagen deposition were noted in the infarcted area, in response to inflammatory cell infiltration and subsequent necrosis of myocardial cells was observed. Therefore, the myocardial fibrosis also affected the surrounding tissue. The current study confirms that aerobic exercise can reduce the activation of inflammatory factors, enhance the reaction of inflammatory cells, reduce the expression of MMP2 and MMP9 protein in rats, and increase the expression of TIMP2. We also found that aerobic exercise can reduce the myocardial CVF following myocardial infarction, and reduce the degree of myocardial fibrosis,this has been consistent with some research findings[23,24]. Therefore, the current study suggests that aerobic exercise can reduce the fibrosis associated with myocardial infarction in the myocardial cells of rats. The mechanism through which this is achieved might be related to the inhibition of inflammatory cytokines, and the regulation of the expression of MMP2, MMP9, and TIMP proteins.

In conclusion, Aerobic exercise can improve myocardial fibrosis after myocardial infarction and promote the expression of netrin-1 and DCC receptors. To the best of our knowledge, this is the first study to explore the relationship between aerobic exercise and the expression of myocardial netrin-1. However, the mechanisms underlying these effects are not yet clear, and warrant further research.

## Supporting information

S1 Data (RAR)
